# Maternity waiting home as a potential intervention for reducing the maternal mortality ratio in El Salvador: an observational case study

**DOI:** 10.1186/s13690-021-00752-8

**Published:** 2021-12-20

**Authors:** Hee sang Yoon, Chong-Sup Kim

**Affiliations:** 1 Institution: Nursing Department, Seoul Women’s College of Nursing, Seoul, Korea; 2 Graduate School of International Studies, Seoul National University, Seoul, Korea

**Keywords:** Maternal mortality ratio, Maternity waiting home, El Salvador

## Abstract

**Background:**

El Salvador is recognized as a country that has effectively reduced its Maternal Mortality Ratio (MMR). While health indicators, such as total fertility rate, adolescent fertility rate, skilled birth attendance, and health expenditures, have improved in El Salvador, this improvement was unremarkable compared to advancements in other developing countries. How El Salvador could achieve an outstanding decrease in MMR despite unexceptional improvements in health and non-health indicators is a question that deserves deep research. We used quantitative methods and an observational case study to show that El Salvador could reduce its MMR more than expected by instituting health policies that not only aimed to reduce the (adolescent) fertility rate, but also provide safe birthing conditions and medical services to pregnant women through maternity waiting homes.

**Methods:**

We ran pooled ordinary least squares regression and panel regression with fixed effects using MMR as the dependent variable and health and non-health factors as the independent variables. We conducted residual analysis, calculated the predicted value of MMR, and compared it with the observed value in El Salvador. To explain the change in MMR in El Salvador, we carried out an observational case study of maternity waiting homes in that country.

**Results:**

El Salvador could reduce MMR by improving health factors such as fertility rate skilled birth attendance and non-health factors, such as gross domestic product (GDP) per capita and female empowerment. However, even while considering these factors, the MMR of El Salvador decreased by more than expected. We confirmed this by analyzing the residuals of the regression model. This improvement in MMR, which is larger than expected from the regression results, can be attributed partly to government measures such as maternity waiting homes.

**Conclusions:**

The reason for the unexplained reduction in El Salvador’s MMR seems to be attributed in part to health policies that not only aim to reduce the fertility rate but also to provide safe birthing conditions and medical services to pregnant women through maternity waiting homes.

**Supplementary Information:**

The online version contains supplementary material available at 10.1186/s13690-021-00752-8.

## Background

El Salvador is recognized as a country that has effectively reduced its maternal mortality ratio (MMR). The Millennium Development Goals (MDG) Report states that the MMR in El Salvador reduced considerably in the 1990 and 2000 s thanks to successful health reforms [1]. The next step is to provide more equitable access to health care services in remote regions and vulnerable populations [2].

The MMR in El Salvador decreased from 118 to 54 per 100,000 live births from 1995 to 2015, which was the largest reduction seen among comparable Latin American countries. The average MMR of Latin American countries decreased from 118 to 1995 to 68 in 2015, a 47.2% reduction. The annual rate of MMR reduction in El Salvador was 5.2% between 1990 and 2015 [3].

While health indicators, such as fertility rate, adolescent fertility rate, skilled birth attendance, and health expenditures, have improved in El Salvador, this improvement was not extraordinary compared with other developing countries. How El Salvador can achieve an outstanding decrease in MMR despite unexceptional improvements in health and non-health indicators is a question that deserves deep research. Our hypothesis is that health policies that not only aim to reduce the (adolescent) fertility rate but also to provide safe birthing conditions and medical services to pregnant women such as maternity waiting homes are the main drivers of reductions in MMR in El Salvador. The MDG report attributes the reduction of MMR to health policies, including the Birth Plan Strategy and Family Health Community Teams, but does not specifically mention maternity waiting homes (MWHs).

Maternity waiting homes are residential facilities located near hospitals where pregnant women can await their delivery and be transferred to the neighboring hospital shortly before giving birth or earlier in case of complications [4]. MWH can increase the facility-based delivery rate by inducing women living in remote areas to give birth at health facilities [5, 6].

The three elements of MWHs are: (i) facilities where pregnant women can comfortably reside before delivery, (ii) policies and financial support, and (iii) easy access to health systems with skilled personnel [7]. Despite some studies that could not find strong evidence of the effectiveness of MWHs, most studies show that MWHs have positive effects on preventing maternal death and stillbirths [5].

In some developing countries, MWHs are an important part of national strategies to improve maternal health services [5]. In Zambia, health authorities have adopted MWHs for decades to overcome demand-side barriers and increase access to skilled birth attendants [6, 7]. In El Salvador, the state of maternal health care services remains poor, particularly for the poorest populations residing in remote areas [8]. The government of El Salvador implemented the MWH program to reduce the maternal mortality ratio in rural areas [2].

To analyze the effects of MWH in El Salvador, we first build an empirical model to explain the differences in MMR across countries. Based on this model, we show that the case of El Salvador cannot be fully explained by the health and non-health indicators traditionally used in MMR analysis. Based on the empirical results, we suggest that the maternity waiting home program was an important factor that partly explains the large drop in MMR in El Salvador.

## Methods

### Characteristics of maternity waiting home

El Salvador is a country that is situated in the southwestern part of Central America. El Salvador’s mountainous geography makes transportation difficult and medical facility accessibility challenging in many regions; transportation services are not readily supported in El Salvador, particularly in the case of expectant mothers in remote areas who need access to hospitals at impending stages before delivery [9, 10].

The Department of Health in El Salvador amended the Medical Service Act in 2010 to strengthen primary medical services and prevent the exclusion of remote residents from medical services [10]. Family Health Community Teams (Unidades Comunitarias de Salud Familiar: UCSF) and maternity waiting homes are important programs for maternal and child health. At the level of municipal health networks, UCSF health centers are responsible for primary health services [9, 10]. Nationwide, there are more than 360 UCSFs that are in charge of maternal and child health [10, 11].

The medical team that works in UCSF is called ECOS (Equipos Comunitarios de Salud: Community Health Team) and consists of doctors, nurses, nutritionists, health education specialists, psychology counsellors, and nurse assistants [10]. ECOS provides primary care at regular check-ups, vaccinations, and medical treatment [12]. Community health workers in UCSF health centers are trained for a certain period of time and placed in a UCSF to learn, research, and routinely report on health problems of the designated region. Furthermore, they keep track of the conditions of expectant mothers in maternal health-related issues and report them to health centers [13]. One community health worker is required to attend to 200 households residing in the region [9].

To strengthen mother and child health services specifically for remote pregnant women [14] and prevent maternal deaths, the government of El Salvador established a program of Maternity Waiting Home for Expectant Mothers in 2009. MWHs are a part of the overall health delivery system in El Salvador [3]. With assistance from the Korea International Cooperation Agency (KOICA) and United States Agency for International Development (USAID), 16 MWHs were set up by 2013.

The objective of the MWH program was to encourage expectant mothers, especially those with high risks, who are spread across remote mountainous areas to seek care and rest at clinics until their due dates when they would be transported to nearby hospitals for delivery [14, 15]. MWHs are located at UCSF or near health centers so that patients can be promptly transferred to hospitals that are equipped with delivery facilities. The majority of expectant mothers enter an MWH approximately 2-3 days before delivery. Although mothers voluntarily come to MWHs, UCSF provides transportation to those who do not have the adequate means of transportation or would have difficulty reaching the MWH. Expectant mothers wait at the MWH until their expected date of delivery and are transferred along with their medical treatment records to a nearby hospital for their delivery via UCSF vehicles, ambulances, or taxis, [9, 14].

When the distance between their residence and the hospital is great and transportation is inefficient, expectant mothers can arrive one day prior to their regular check-up, and remain at the MWH, and return home after receiving treatment [9, 16, 17]. After delivery, mothers can also return to the MWH and receive vaccinations for newborn babies. Furthermore, MWH health clinics act as safe havens for expectant mothers and newborn babies facing domestic violence.

All services provided at MWHs are free of charge. The management budget for each MWH is provided by the Department of Health even if there are cases in which a part of the budget is sponsored by regional steering committees or sponsors. During their stay at an MWH, expectant mothers are provided with meals and treatments that are particularly essential during pregnancy. After delivery, mothers are provided with clothing for their newborn babies [9, 15].

At the MWH, licensed specialists in maternal and child health are available on-site 24/7 and manage the health conditions of both expectant mothers and fetuses. Specialists check the health of expectant mothers and listening to cardiac sounds of the fetus twice a day. Personnel at MWHs provide health education on reproductive health and delivery to expectant mothers [14]. The government designates approximately 40% of the expectant mothers in remote areas as the target population of the MWH program [9]. The remaining 60% of expectant mothers are considered to have access to means of transportation to hospitals for delivery and do not require MWH services. Admission to MWHs takes place through recommendations from community health workers, UCSFs, or referrals (i.e., mothers who have previously experienced the MWH).

### Study design

Even if MWHs are important to minimizing the MMR in El Salvador, they cannot be considered in regression analysis as no data exist. In this situation, our research strategy is to run a regression using health and non-health variables that are commonly used to explain MMR and then analyze how much of the MMR reduction in El Salvador can be attributed to these variables versus how much can be attributed to other reasons that are specific to El Salvador. We can do this by analyzing the residuals, i.e., the unexplained part, in the regression.

### Data source

We used data from the World Development Indicators (WDI), which provides cross-country data on development [18]. WDI is a publicly available data base in the World Bank.

There were 143 countries included in the regression and the years under consideration were from 2000 to 2015 [18].

### Selection of variables

The dependent variable was the maternal mortality ratio per 100,000 live births (MMR). Control variables were taken from the literature and divided into two categories: (1) health sector variables and (2) non-health-related variables [19]. The variables in the health sector are total fertility rate (TFR) measured as births per woman, adolescent fertility rate (AFR) measured by births per 1,000 women ages 15-19, percent of births attended by skilled health staff (SKILL), and current health expenditure (HEATH) measured by percent of GDP. The non-health variables include those that may affect maternal health, which are gross domestic product per capita (GDPC) expressed in U.S. dollars, female secondary school enrollment rate (FSECOND) expressed as net percent, percentage of the rural population that has access to electricity (ELECTRIC), urban population (URBAN) as a percent of the total population, female labor force participation rate (FPARTICI) measured as percent of the female population ages 15^+^ in the labor force), and percentage of seats held by women in national parliaments (FPARLIA). The variables included in the model are explained in Table 1.

**Table 1:** Variables included in the regression model of MMR

	Variables	Explanation	Source
Dependent Variable	MMR	Maternal mortality ratio (per 100,000 live births)	WDI
Independent Variables (Health Sector)	TFR	Total Fertility Rate (births per woman)	WDI
	AFR	Adolescent Fertility Rate (births per 1,000 women ages 15-19)	WDI
	SKILL	Births attended by skilled health staff (% of total)	WDI
	HEALTH	Current health expenditure (% of GDP)	WDI
Independent Variables (Non-Health Sector)	GDPC	GDP per capita (dollars)	WDI
	FSECOND	School enrollment, secondary, female (% net)	WDI
	ELECTRIC	Access to electricity, rural (% of rural population)	WDI
	URBAN	Urban population (% of total population)	WDI
	FPARTICI	Labor force participation rate, female (% of female population ages 15 +)	WDI
	FPARLIA	Proportion of seats held by women in national parliaments (%)	WDI

Source: World Development Indicators (WDI)

### Regression

For our empirical methods, we applied pooled ordinary least squares (OLS) regression to analyze the effects of the independent variables on MMR, and panel regression with fixed effects, where time-invariant country-specific characteristics are included.

The regression model is the following:

$${log}(MMR{)}_{it}=\alpha +{\beta }_{1}{log}(TFR{)}_{it}+{\beta }_{2}{log}(AFR{)}_{it}+{\beta }_{3}{log}(SKILL{)}_{it}+{\beta }_{4}{log}(HEALTH{)}_{it}+{\beta }_{5}{log}(GDPC{)}_{it}+{\beta }_{6}{log}(FSECOND{)}_{it}+{\beta }_{7}{log}(ELECTRIC{)}_{it}+{\beta }_{8}{log}(URBAN{)}_{it}+{\beta }_{9}{log}(FPARTICI{)}_{it}+{\beta }_{10}log(FPARLIA{)}_{it}+({\mu }_{i})+{\epsilon }_{it}$$

where ${\mu }_{i}$ is the fixed effect used only in the panel regression, which captures the effects of time-invariant country-specific characteristics. After the regression, we performed an analysis of the residuals, which are the differences between the observed values of the dependent variable and the predicted values. We calculated the predicted value of El Salvador based on the fixed effect panel regression model and compared it with the observed value as follows:

Residual (*e*) = Observed value (*y*) - Predicted value (*ŷ*).

Finally, we carried out a case study of maternity waiting homes in El Salvador to investigate the unexplained reduction in MMR.

### Ethical considerations

This study was granted a waiver of review by the Institutional Review Board of Seoul Women’s College of Nursing (No. SWCN-201407-HR-002). The need for informed consent was waivered by the Institutional Review Board of Seoul Women’s College of Nursing.

### Results of Pooled OLS regression

The results of the regressions are shown in Table 2. In the pooled OLS regression, the coefficients have the correct signs and are mostly significant. From the results, it can be inferred that if the total fertility rate decreases by 1%, then MMR would decrease by 0.97%. If the adolescent fertility rate decreases by 1%, then MMR would decrease by 0.49%. If skilled birth attendance or health expenditure increases, then MMR would decrease. Among the non-health sector indicators, GDP per capita appears to be the most important variable. If GDP per capita increases by 1%, then MMR would decrease by 0.30%. Improvements in female secondary school enrollment, access to electricity, and women’s empowerment also reduce MMR with different levels of significance.

**Table 2:** Regression results

		pooled OLS		Panel regression (FE)	
Variables		Coef.	C.I.(95%)	Coef.	C.I.(95%)
Health Sector	log(TFR)	0.97^**^	0.82 ~ 1.12	-0.33	-0.70 ~ 0.04
	log(AFR)	0.49^**^	0.42 ~ 0.56	0.48^**^	0.27 ~ 0.69
	log(SKILL)	-0.12	-0.26 ~ 0.02	-0.18^**^	-0.31 ~ -0.06
	log(HEALTH)	-0.16^**^	-0.28 ~ -0.04	-0.06	-0.19 ~ 0.08
Non-Health Sector	log(GDPC)	-0.30^**^	-0.35 ~ -0.24	-0.46^**^	-0.68 ~ -0.23
	log(FSECOND)	-0.13	-0.30 ~ 0.05	-0.05	-0.16 ~ 0.06
	log(ELECTRIC)	-0.10^**^	-0.17 ~ -0.03	-0.02	-0.05 ~ 0.02
	log(URBAN)	-0.15^*^	-0.29 ~ -0.01	-0.22	-0.74 ~ 0.30
	log(FPARTICI)	0.06	-0.09 ~ 0.21	-0.21	-0.44 ~ 0.02
	log(FPARLIA)	-0.11^**^	-0.17 ~ -0.05	-0.00	-0.06 ~ 0.05
	Constant	4.21^**^	3.26 ~ 5.16	5.99^**^	3.68 ~ 8.29
		N. obs=982 R²= 0.857		N. obs= 982 N. groups=143 R² within = 0.531 between = 0.789 overall = 0.756	

*Significant at 0.05; **Significant at 0.01

### Results of panel regression with fixed effects

In the panel regression with fixed effects such that the time-invariant heterogeneity of the countries is controlled, even if the total fertility rate loses significance, the adolescent fertility rate is still significant, and the coefficient has a similar value as that in the pooled OLS. This means that for an individual country, reducing the adolescent fertility rate is much more important than reducing the total fertility rate to reduce MMR.

Skilled birth attendance has the correct sign and is significant. Among the non-health sector indicators, GDP per capita is the only significant indicator. The other variables are not significant, which may be because their respective intra-country variation is insufficient to explain the change in MMR within a given country, even if these variables can explain inter-country differences in MMR. The model explains 53.1% of the MMR variation within a country as shown by the “within” R^2^. Overall, the most important variable that explains the change in MMR within a country appears to be the adolescent fertility rate (Table 2).

### Residual analysis in regression

**Fig. 1:**
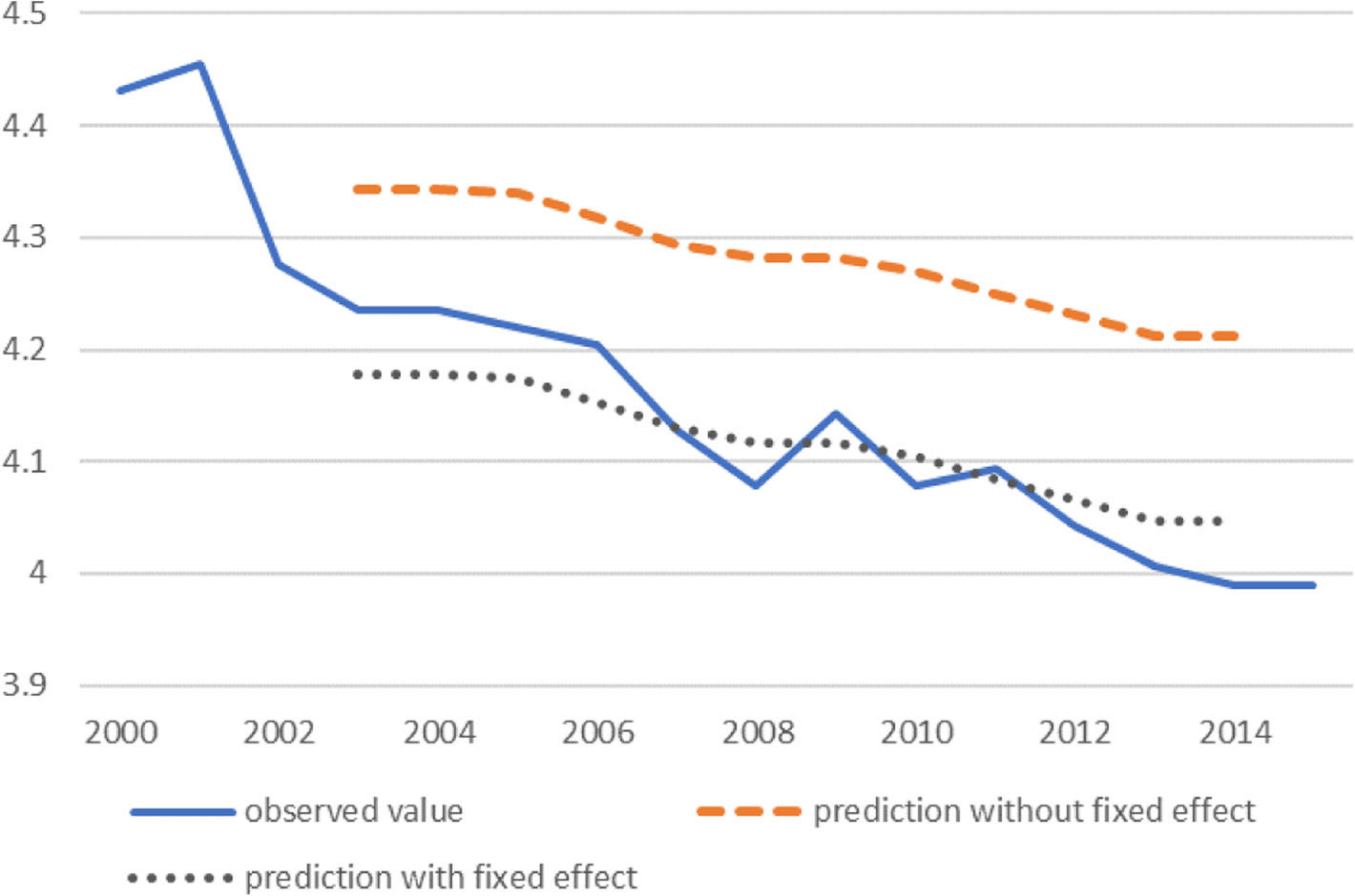
MMR in El Salvador

In our analysis, we calculated the predicted value of El Salvador based on the fixed effect panel regression model and compared it with the observed value. In Fig. 1, prediction without fixed effects is the predicted value of MMR considering the value of the independent variables but not excluding the fixed effect. This predicted value is much higher than the observed value. Prediction without fixed effects decreased as variables such as adolescent fertility rate, skilled birth attendance, and GDP per capita in El Salvador improved during the period of analysis. However, this fails to consider the existence of unobserved time invariant characteristics of El Salvador. Even fully considering the independent variables, El Salvador had a consistently lower MMR than expected. If the negative fixed effect is included, then the predicted value is lower as shown by the prediction including the fixed effect in Fig. 1. This negative fixed effect may be attributed to an efficient health care system in El Salvador, which is not reflected in the independent variables. The graph shows that the observed value decreased more rapidly than the prediction with fixed effects, resulting in increased residuals. This trend cannot be explained by either independent variables or fixed effects. Therefore, this trend must be explained by other changes in policies or health systems in El Salvador during this period, which are not reflected in the independent variables. Although there were large drops in MMR in 2002, 2007, and 2008, we would like to focus on the decline after 2011 (Fig. 1).

The MMR in El Salvador decreased from 118 to 54 100,000 live births from 1995 to 2015, a reduction of 54.2%. This was the largest reduction among comparable Latin American countries. The average MMR of Latin American countries decreased from 118 to 1995 to 68 in 2015, a 42.4% reduction. El Salvador has met the MDG targets regarding MMR. Despite an outstanding reduction in MMR, other health indicators did not improve on the same scale. The total fertility rate in El Salvador declined from 3.58 to 1995 to 2.10 in 2015, which is not as remarkable as that of other Latin American countries. The reduction in the adolescent fertility rate was 27.2%, which is slightly higher than that of other Latin American countries. In contrast, the health expenditure to GDP ratio declined by 0.9% points, exerting a negative impact on MMR.

**Fig. 2:**
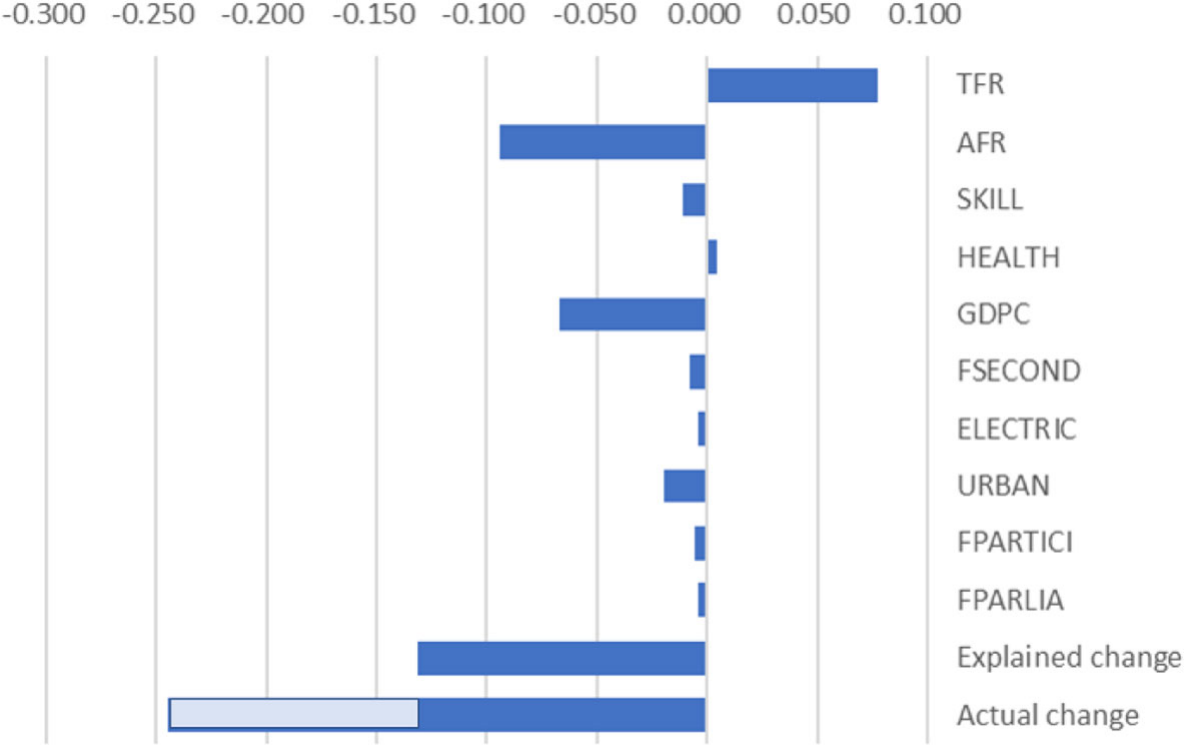
Factor contributing to the reduction of MMR in El Salvador

The contribution of each variable to the reduction in MMR between 2003 and 2014 is shown in Fig. 2. The largest contribution came from the reduction in the adolescent fertility rate and the increase in GDP per capita.

However, the variables included in the regression only explain approximately half of the reduction in MMR. The other half must be explained by other particular characteristics of El Salvador that were not considered in the regression analysis. Health policy in El Salvador was not included in the regression analysis, as there is no proper measurement for the 143 countries included in the regression. Its effect will be partly reflected in the fixed effect and residuals. We suggest that health policy in El Salvador, including the MWH program, is a particular characteristic of El Salvador that contributed to the unexplained reduction of MMR.

### MWH and maternal deaths in El Salvador

The role of the MWH program is to prevent maternal deaths as a result of pregnancy rather than to prevent the pregnancy itself. Therefore, MWH contributed to the reduction of MMR in El Salvador by providing medical service to pregnant adolescents. This effect is not reflected in the explanatory variables of the regression due to data limitations and therefore must be considered as part of the fixed effect or residuals.

In 2013, the total number of MWH patients was 2,587. Among them, 913 patients were adolescents. The share of adolescents among MWH patients was 35% (Table 3). This implies that even with preventive education, many adolescents will still become pregnant and be at risk of maternal death. Adolescents may receive MWH services and care once they are pregnant. Until now, there have been no reported maternal deaths among adolescent patients in MWHs.

**Table 3:** El Salvador Nationwide MWH Number of Users (2013)

	Adolescents up to 19 years old	Adults over 20 years old	Total
Atiquizaya	59	118	177
Corinto	83	164	247
Suchitoto	9	18	27
Cara Sucia	26	52	78
Coatepeque	74	111	185
Sonsonate	101	243	344
La Palma	64	144	208
Colón	64	97	161
La Libertad	23	33	56
San Juan Nonualco	42	61	103
La Herradura	31	44	75
Panchimalco	22	31	53
San Gerardo	17	38	55
Perquin	120	234	354
Anamoros	57	107	164
La Union	121	179	300
Total	913	1,674	2,587

Source: KOICA

## Discussion

Health factors, such as the total fertility rate, adolescent fertility rate, and health expenditure, are important determinants of MMR [19, 20]. If these factors improve, we would expect an improvement in the MMR. Naturally, with an outstanding improvement in MMR, we would expect an equal improvement of these other health. This was not the case for El Salvador; an outstanding improvement in MMR was accompanied by a moderate improvement in health indicators and a decrease in health expenditure.

One of the most important variables that explains the change in the MMR is the adolescent fertility rate. The importance of the adolescent fertility rate has been pointed out by many studies [20–22]. According to UNFPA [21] and WHO and UNFPA [22], adolescents aged 15 through 19 are twice as likely to die during pregnancy or childbirth as those over age 20; similarly, girls under age 15 are five times more likely to die [20–22]. However, MMR decreased in El Salvador by more than expected from the reduction in the adolescent fertility rate, but this decrease was less than that of similar countries. We attribute this result to successful efforts in El Salvador to prevent pregnancies among adolescents and even greater success in preventing maternal deaths among adolescents during or after the delivery of the baby. We suggest that this partial success can be explained by UCSF and MWH programs.

The 3rd report on the Progress of the Millennium Development Goals of El Salvador states [1]:

*Non-hospital maternal mortality, in particular, has been occurring with decreasing frequency. Healthcare representatives and, more recently, Family Health Community Teams successfully managed to implement the Birth Plan strategy, mainly in rural communities, with the primary purpose of encouraging women and their families to take any necessary steps to give birth at the hospital. This has made it possible today for more than 90% of births to take place in hospitals, mainly belonging to the public network and the Salvadoran Institute of Social Insurance.*

In 2013, expectant mothers’ average use of MWH throughout the seven MWHs was approximately 70%, which is close to the government’s goal of 80% coverage. El Salvador’s mountainous geography makes transportation difficult and medical facility accessibility challenging in many regions; transportation services are not readily supported in El Salvador, particularly in the case of expectant mothers in remote areas who need to access hospitals at the impending stages before delivery. MWHs were constructed to serve the medical needs of expectant mothers from remote areas. It appears that MWHs have increased facility-based deliveries and reduced maternal and child mortality rates in remote areas.

It is clear that the UCSF and MWH systems have reduced MMR by connecting vulnerable expectant mothers to hospitals and enabling facility-based delivery [5, 6]. Since the most vulnerable group of expectant mothers are adolescents [8], the system inarguably reduced the number of maternal deaths among adolescents [4]. Similar effects were found in Nepal, where MWHs reduced maternal deaths among adolescents [23, 24]. The extremely high proportion of adolescent patients may be explained by the high rates of adolescent pregnancy in remote and rural areas [9, 25, 26], the heavy reliance of adolescents on this system, and the effective efforts of UCSF in guiding pregnant adolescents to MWHs [8–10]. However, the system was not successful in reducing pregnancy among adolescents. Adolescents were provided education about sex and maternal care once they were admitted to MWHs (therefore, not before becoming pregnant). Of course, there is some degree of sexual education in school curricula, but these efforts are evidently ineffective in reducing adolescent pregnancy [27].

One limitation of this study is that by using country-level data, we could not employ MWH as an independent variable in the regression. Cluster randomized controlled trials would be a viable solution to address this limitation. To do so, we need more granular data, such as TFR or MMR by region or village. If such data exist, then we could use the time variation as MWHs were installed across different regions and compare the MMR.

## Conclusions

We have shown that El Salvador could reduce its MMR by improving health factors, such as total fertility rate, adolescent fertility rate, skilled birth attendance, and non-health factors, such as GDP per capita and women’s empowerment. However, even considering these factors, the MMR of El Salvador decreased by more than expected. We confirmed this by analyzing the residuals of a regression model. The reason for this unexplained reduction in El Salvador’s MMR appears to be attributable to health policies that not only aimed to reduce the (adolescent) fertility rate but also provided safe birthing conditions and medical services to pregnant women such as MWHs. Nevertheless, as the adolescent fertility rate is still too high and pregnancy among adolescents continues to be a significant driver of maternal deaths, more effective policies to reduce adolescent pregnancies are needed.

## Supplementary information

**Additional file 1.**

**Additional file 2.**

## Data Availability

The datasets used and analyzed during the current study are available from the corresponding author.

## Abbreviations

AFR: Adolescent Fertility Rate (births per 1,000 women ages 15-19)
Coef: coefficient
ECOS: Equipos Comunitarios de Salud
ELECTRIC: Access to electricity, rural (% of rural population)
FE: Fixed Effect
FPARLIA: Proportion of seats held by women in national parliaments (%)
FPARTICI: Female Labor Force Participation %
FSECOND: School enrollment, secondary, female (% gross)
HEM: Hogar de Espera Materna(Maternity Waiting Home in Spanish)
GDPC: GDP per capita (dollars)
HEALTH: Total health expenditure to GDP ratio (%)
MWH: Maternity Waiting Home
MDGs: Millennium Development Goals
MMR: Maternal mortality ratio (per 100,000 live births)
Obs: Observation
SKILL: Skilled Birth Attendance (% of total)
TFR: Total Fertility Rate (births per woman)
UCSF: Unidades Comunitarias de Salud Familiar
URBAN: Urban population (% of total)
WDI: World Development Indicators
