# Supplementary material for: Maternity waiting home as a potential intervention for reducing the maternal mortality ratio in El Salvador: an observational case study
Source: Arch Public Health. 2021 Dec 20;79:228. doi: 10.1186/s13690-021-00752-8 (PMC8690890; doi:10.1186/s13690-021-00752-8)
Supplement: Supplementary file 1 — Additional file 1. [file 13690_2021_752_MOESM1_ESM.docx]

**Maternity Waiting Home as a Potential Intervention for Reducing the Maternal Mortality Ratio in El Salvador: An Observational Case Study**

Hee sang Yoon ^1*^, Chong sup Kim^2^

1 Institution: Nursing Department, Seoul Women’s College of Nursing, Seoul, Korea

2 Graduate School of International Studies, Seoul National University, Seoul, Korea

**^*^** Corresponding author Hee sang Yoon [viva826@snjc.ac.kr](mailto:viva826@snjc.ac.kr)

**Abstract:**

Background: El Salvador is recognized as a country that has effectively reduced its Maternal Mortality Ratio (MMR). While health indicators, such as total fertility rate, adolescent fertility rate, skilled birth attendance, health expenditures, have improved in El Salvador, this improvement was unremarkable compared to advancements in other developing countries. How El Salvador could achieve an outstanding decrease in MMR despite unexceptional improvement in health and non-health indicators is a question that deserves a deep research. We used quantitative methods and an observational case study to show that the reason El Salvador could reduce MMR more than expected is the health policy that El Salvador could reduce its MMR more than expected by instituting health policies that not only aimed to reduce the (adolescent) fertility rate, but also provided a safe birthing conditions and medical services to pregnant women through maternity waiting homes.

Methods: We ran pooled ordinary least squares regression and panel regressions with fixed effects using MMR as the dependent variable and health and non-health factors as the independent variables. We conducted residual analysis, calculated the predicted value of MMR, and compared it with the observed value in El Salvador. To explain the change of MMR in El Salvador, we carried out an observational case study of maternity waiting home in that country.

Results: El Salvador could reduce MMR by improving health factors such as fertility rate, skilled birth attendance, and non-health factors, such as gross domestic product (GDP) per capita and female empowerment. However, even while considering these factors, the MMR of El Salvador decreased by more than expected. We confirmed this by analyzing the residuals of the regression model. This improvement in MMR, which is larger than expected from the regression results, can be attributed partly to the government measures, such as maternity waiting homes.

Conclusions: The reason for the unexplained reduction in El Salvador’s MMR seems to be attributed in part to health policies that not only aimed to reduce the fertility rate but also to provide safe birthing conditions and medical services to pregnant women through maternity waiting homes.

Key words: Maternal mortality ratio; Maternity waiting home; El Salvador

**Background**

El Salvador is recognized as a country that has effectively reduced its maternal mortality ratio (MMR). The Millennium Development Goals (MDG) Report states that the MMR in El Salvador reduced considerably in the 1990s and 2000s thanks to successful health reforms [1]. The next step is to provide more equitable access to health care services in remote regions and vulnerable populations [2].

The MMR in El Salvador decreased from 118 to 54 per 100,000 live births from 1995 to 2015, which was the largest reduction seen among comparable Latin American countries. The average MMR of Latin American countries decreased from 118 in 1995 to 68 in 2015, a 47.2% reduction. The annual rate of MMR reduction in El Salvador was 5.2% between 1990 and 2015 [3].

While health indicators, such as fertility rate, adolescent fertility rate, skilled birth attendance, and health expenditures, have improved in El Salvador, this improvement was not extraordinary compared with other developing countries. How El Salvador can achieve an outstanding decrease in MMR despite unexceptional improvements in health and non-health indicators is a question that deserves deep research. Our hypothesis is that health policies that not only aim to reduce the (adolescent) fertility rate but also to provide safe birthing conditions and medical services to pregnant women such as maternity waiting homes are the main drivers of reductions in MMR in El Salvador. The MDG report attributes the reduction of MMR to health policies, including the Birth Plan Strategy and Family Health Community Teams, but does not specifically mention maternity waiting homes (MWHs).

Maternity waiting homes are residential facilities located near hospitals where pregnant women can await their delivery and be transferred to the neighboring hospital shortly before giving birth or earlier in case of complications [4]. MWH can increase the facility-based delivery rate by inducing women living in remote areas to give birth at health facilities [5, 6].

The three elements of MWHs are: i) facilities where pregnant women can comfortably reside before delivery, ii) policies and financial support, and iii) easy access to health systems with skilled personnel [7]. Despite some studies that could not find strong evidence of the effectiveness of MWHs, most studies show that MWHs have positive effects on preventing maternal death and stillbirths [5].

In some developing countries, MWHs are an important part of national strategies to improve maternal health services [5]. In Zambia, health authorities have adopted MWHs for decades to overcome demand-side barriers and increase access to skilled birth attendants [6, 7]. In El Salvador, the state of maternal health care services remains poor, particularly for the poorest populations residing in remote areas [8]. The government of El Salvador implemented the MWH program to reduce the maternal mortality ratio in rural areas [2].

To analyze the effects of MWH in El Salvador, we first build an empirical model to explain the differences in MMR across countries. Based on this model, we show that the case of El Salvador cannot be fully explained by the health and non-health indicators traditionally used in MMR analysis. Based on the empirical results, we suggest that the maternity waiting home program was an important factor that partly explains the large drop in MMR in El Salvador.

**Methods**

**Characteristics of maternity waiting home**

El Salvador is a country that is situated in the southwestern part of Central America. El Salvador’s mountainous geography makes transportation difficult and medical facility accessibility challenging in many regions; transportation services are not readily supported in El Salvador, particularly in case of expectant mothers in remote areas who need access to hospitals at impending stages before delivery [9,10].

The Department of Health in El Salvador amended the Medical Service Act in 2010 to strengthen primary medical services and prevent exclusion of remote residents in medical services [10]. Family Health Community Teams (Unidades Comunitarias de Salud Familiar: UCSF) and maternity waiting homes are important programs for maternal and child health. At the level of municipal health networks, UCSF health centers are responsible for primary health services [9,10]. Nationwide, there are more than 360 UCSF that are in charge of maternal and child health [10,11].

The medical team that works in UCSF is called ECOS (Equipos Comunitarios de Salud: Community Health Team), and consists of doctors, nurses, nutritionists, health education specialists, psychology counsellors, and nurse assistants [10]. ECOS provides primary care on regular check-up, vaccination, and medical treatment [12]. Community health worker in UCSF health centers are trained for a certain period of time and placed in UCSF to learn, research, and routinely report on health problems of the designated region. Furthermore, they keep track of conditions of expectant mothers in maternal health- related issues and report them to health centers [13]. One community health worker is required to attend to 200 households residing in the region [9].

To strengthen mother and child health services specifically for remote pregnant women [14] and preventing maternal deaths, the government of El Salvador established a program of Maternity Waiting Home for Expectant Mothers in 2009. MWHs are a part of the overall health delivery system in El Salvador [3]. With assistance from the Korea International Cooperation Agency (KOICA) and United States Agency for International Development (USAID), 16 MWHs were set up by 2013.

The objective of the MWH program was to encourage expectant mothers, especially those with high risks, who are spread across remote mountainous areas to seek care and rest at clinics until their due dates when they would be transported to nearby hospitals for delivery [14, 15]. MWHs are located at UCSF or near health centers so that patients can be promptly transferred to hospitals that are equipped with delivery facilities. The majority of expectant mothers enter an MWH approximately 2-3 days before delivery. Although mothers voluntarily come to MWHs, UCSF provides transportation to those who do not have the adequate means of transportation or would have difficulty reaching the MWH. Expectant mothers wait at the MWH until their expected date of delivery and are transferred along with their medical treatment records to a nearby hospital for their delivery via UCSF vehicles, ambulances, or taxis, [9, 14].

When the distance between their residence and the hospital is great and transportation is inefficient, expectant mothers can arrive one day prior to their regular check-up, and remain at the MWH, and return home after receiving treatment [9,16,17]. After delivery, mothers can also return to the MWH and receive vaccinations for newborn babies. Furthermore, MWH health clinics act as safe havens for expectant mothers and newborn babies facing domestic violence.

All services provided at MWHs are free of charge. The management budget for each MWH is provided by the Department of Health even if there are cases in which a part of the budget is sponsored by regional steering committees or sponsors. During their stay at an MWH, expectant mothers are provided with meals and treatments that are particularly essential during pregnancy. After delivery, mothers are provided with clothing for their newborn babies [9, 15].

At the MWH, licensed specialists in maternal and child health are available on-site 24/7 and manage the health conditions of both expectant mother and fetuses. Specialists check the health of expectant mothers and listening to cardiac sounds of the fetus twice a day. Personnel at MWHs provide health education on reproductive health and delivery to expectant mothers [14]. The government designates approximately 40% of the expectant mothers in remote areas as the target population of the MWH program [9]. The remaining 60% of expectant mothers are considered to have access to means of transportation to hospitals for delivery and do not require MWH services. Admission to MWHs takes place through recommendations from community health workers, UCSFs, or referrals (i.e., mothers who have previously experienced the MWH).

**Study design**

Even if MWHs are important to minimizing the MMR in El Salvador, they cannot be considered in regression analysis as no data exist. In this situation, our research strategy is to run a regression using health and non-health variables that are commonly used to explain MMR and then analyze how much of the MMR reduction in El Salvador can be attributed to these variables versus how much can be attributed to other reasons that are specific to El Salvador. We can do this by analyzing the residuals, i.e., the unexplained part, in the regression.

**Data sources**

We used data from the World Development Indicators (WDI), which provides cross-country data on development [18]. WDI is a publicly available data base in the World Bank.

There were 143 countries included in the regression and the years under consideration were from 2000 to 2015 [18].

**Selection of variables**

The dependent variable was the maternal mortality ratio per 100,000 live births (MMR). Control variables were taken from the literature, and divided into two categories: 1) health sector variables and 2) non-health-related variables [19]. The variables in the health sector are total fertility rate (TFR) measured as births per woman, adolescent fertility rate (AFR) measured by births per 1,000 women ages 15-19, percent of births attended by skilled health staff (SKILL) , and current health expenditure (HEATH) measured by percent of GDP. The non-health variables include those that may affect maternal health, which are gross domestic product per capita (GDPC) expressed in U.S. dollars, female secondary school enrollment rate (FSECOND) expressed as net percent, percentage of the rural population that has access to electricity (ELECTRIC), urban population (URBAN) as a percent of the total population, female labor force participation rate (FPARTICI) measured as percent of the female population ages 15^+^ in the labor force) , and percentage of seats held by women in national parliaments (FPARLIA). The variables included in the model are explained in Table 1.

**Data analysis**

For our empirical methods, we applied pooled ordinary least squares (OLS) regression to analyze the effects of the independent variables on MMR, and panel regression with fixed effects, where time-invariant country-specific characteristics are included.

The regression model is the following:

$$\log(MMR)_{it}=\alpha+\beta_{1}\log(TFR)_{it}+\beta_{2}\log(AFR)_{it}+\beta_{3}\log(SKILL)_{it}+\beta_{4}\log(HEALTH)_{it}+\beta_{5}\log(GDPC)_{it}+\beta_{6}\log(FSENCOND)_{it}+\beta_{7}\log(ELECTRIC)_{it}+\beta_{8}\log(URBAN)_{it}+\beta_{9}\log(FPARTICI)_{it}+\beta_{10}\log(FPARLIA)_{it}+(\mu_{i})+\varepsilon_{it}$$

where $\mu_{i}$ is the fixed effect used only in the panel regression, which captures the effects of time-invariant country-specific characteristics. After the regression, we performed an analysis of the residuals, which are the differences between the observed values of the dependent variable and the predicted values. We calculated the predicted value of El Salvador based on the fixed effect panel regression model and compared it with the observed value as follows:

$$\log\left( MMR \right)it=\alpha+\beta_{1}\log\left( TFR \right)it+\beta_{2}\log\left( AFR \right)it+\beta_{3}\log\left( SKILL \right)it+\beta_{4}\log\left( HEALTH \right)it+\beta_{5}\log\left( GDPC \right)it+\beta_{6}\log\left( FSENCOND \right)it+\beta_{7}\log\left( ELECTRIC \right)it+\beta_{8}\log\left( URBAN \right)it+\beta_{9}\log\left( FPARTICI \right)it+\beta_{10}\log\left( FPARLIA \right)it+\varepsilon it$$

$$\log\left( MMR \right)it=\alpha+\beta_{1}\log\left( TFR \right)it+\beta_{2}\log\left( AFR \right)it+\beta_{3}\log\left( SKILL \right)it+\beta_{4}\log\left( HEALTH \right)it+\beta_{5}\log\left( GDPC \right)it+\beta_{6}\log\left( FSENCOND \right)it+\beta_{7}\log\left( ELECTRIC \right)it+\beta_{8}\log\left( URBAN \right)it+\beta_{9}\log\left( FPARTICI \right)it+\beta_{10}\log\left( FPARLIA \right)it+\mu i+\varepsilon it$$

Residual (e) = Observed value (y) - Predicted value (ŷ)

Finally, we carried out a case study of maternity waiting home in El Salvador to investigate the unexplained reduction in MMR.

**Ethical consideration**

This study was granted a waiver of review by the Institutional Review Board of Seoul Women’s College of Nursing (No. SWCN-201904-HR-003). The need for informed consent was waivered by the Institutional Review Board of Seoul Women’s College of Nursing.

**Results of Pooled OLS regression**

The results of the regressions are shown in Table 3. In the pooled OLS regression, the coefficients have the correct signs and are mostly significant. From the results, it can be inferred that if the total fertility rate decreases by 1%, then MMR would decrease by 0.97%. If the adolescent fertility rate decreases by 1%, then MMR would decrease by 0.49%. If skilled birth attendance or health expenditure increases, then MMR would decrease. Among the non-health sector indicators, GDP per capita appears to be the most important variable. If GDP per capita increases by 1%, then MMR would decrease by 0.30%. Improvements in female secondary school enrollment, access to electricity, and women’s empowerment also reduce MMR with different levels of significance.

**Results of panel regression with fixed effects**

In the panel regression with fixed effects such that the time-invariant heterogeneity of the countries is controlled, even if the total fertility rate loses significance, the adolescent fertility rate is still significant, and the coefficient has a similar value as that in the pooled OLS. This means that for an individual country, reducing the adolescent fertility rate is much more important than reducing the total fertility rate to reduce MMR.

Skilled birth attendance has the correct sign and is significant. Among the non-health sector indicators, GDP per capita is the only significant indicator. The other variables are not significant, which may be because their respective intra-country variation is insufficient to explain the change in MMR within a given country, even if these variables can explain inter-country differences in MMR. The model explains 53.1% of the MMR variation within a country as shown by the “within” R^2^. Overall, the most important variable that explains the change in MMR within a country appears to be the adolescent fertility rate (Table 2).

< Table 2 >

**Residual analysis in regression**

In our analysis, we calculated the predicted value of El Salvador based on the fixed effect panel regression model and compared it with the observed value. In Figure 1, prediction without fixed effects is the predicted value of MMR considering the value of the independent variables but not excluding the fixed effect. This predicted value is much higher than the observed value. Prediction without fixed effects decreased as variables such as adolescent fertility rate, skilled birth attendance, and GDP per capita in El Salvador improved during the period of analysis. However, this fails to consider the existence of unobserved time invariant characteristics of El Salvador. Even fully considering the independent variables, El Salvador had a consistently lower MMR than expected. If the negative fixed effect is included, then the predicted value is lower as shown by the prediction including the fixed effect in Figure 1. This negative fixed effect may be attributed to an efficient health care system in El Salvador, which is not reflected in the independent variables. The graph shows that the observed value decreased more rapidly than the prediction with fixed effects, resulting in increased residuals. This trend cannot be explained by either independent variables or fixed effects. Therefore, this trend must be explained by other changes in policies or health systems in El Salvador during this period, which are not reflected in the independent variables. Although there were large drops in MMR in 2002, 2007, and 2008, we would like to focus on the decline after 2011 (Figure 1).

< Figure 1 >

The MMR in El Salvador decreased from 118 to 54 100,000 live births from 1995 to 2015, a reduction of 54.2%. This was the largest reduction among comparable Latin American countries. The average MMR of Latin American countries decreased from 118 in 1995 to 68 in 2015, a 42.4% reduction. El Salvador has met the MDG targets regarding MMR. Despite an outstanding reduction in MMR, other health indicators did not improve on the same scale. The total fertility rate in El Salvador declined from 3.58 in 1995 to 2.10 in 2015, which is not as remarkable as that of other Latin American countries. The reduction in the adolescent fertility rate was 27.2%, which is slightly higher than that of other Latin American countries. In contrast, the health expenditure to GDP ratio declined by 0.9 percentage points, exerting a negative impact on MMR.

The contribution of each variable to the reduction in MMR between 2003 and 2014 is shown in Figure 2. The largest contribution came from the reduction in the adolescent fertility rate and the increase in GDP per capita.

< Figure 2>

However, the variables included in the regression only explain approximately half of the reduction in MMR. The other half must be explained by other particular characteristics of El Salvador that were not considered in the regression analysis. Health policy in El Salvador was not included in the regression analysis, as there is no proper measurement for the 143 countries included in the regression. Its effect will be partly reflected in the fixed effect and residuals. We suggest that health policy in El Salvador, including the MWH program, is a particular characteristic of El Salvador that contributed to the unexplained reduction of MMR.

**MWH and maternal deaths in El Salvador**

The role of the MWH program is to prevent maternal deaths as a result of pregnancy rather than to prevent the pregnancy itself. Therefore, MWH contributed to the reduction of MMR in El Salvador by providing medical service to pregnant adolescents. This effect is not reflected in the explanatory variables of the regression due to data limitations and therefore must be considered as part of the fixed effect or residuals.

In 2013, the total number of MWH patients was 2,587. Among them, 913 patients were adolescents. The share of adolescents among MWH patients was 35% (Table 3). This implies that even with preventive education, many adolescents will still become pregnant and be at risk of maternal death. Adolescents may receive MWH services and care once they are pregnant. Until now, there have been no reported maternal deaths among adolescent patients in MWHs.

< Table 3 >

**Discussion**

Health factors, such as the total fertility rate, adolescent fertility rate, and health expenditure, are important determinants of MMR [19, 20]. If these factors improve, we would expect an improvement in the MMR. Naturally, with an outstanding improvement in MMR, we would expect an equal improvement of these other health. This was not the case for El Salvador; an outstanding improvement in MMR was accompanied by a moderate improvement in health indicators and a decrease in health expenditure.

One of the most important variables that explains the change in the MMR is the adolescent fertility rate. The importance of the adolescent fertility rate has been pointed out by many studies [20-22]. According to UNFPA [21] and WHO and UNFPA [22], adolescents aged 15 through 19 are twice as likely to die during pregnancy or childbirth as those over age 20; similarly, girls under age 15 are five times more likely to die [20-22]. However, MMR decreased in El Salvador by more than expected from the reduction in the adolescent fertility rate, but this decrease was less than that of similar countries. We attribute this result to successful efforts in El Salvador to prevent pregnancies among adolescents and even greater success in preventing maternal deaths among adolescents during or after the delivery of the baby. We suggest that this partial success can be explained by UCSF and MWH programs.

The 3^rd^ report on the Progress of the Millennium Development Goals of El Salvador states [1]:

*Non-hospital maternal mortality, in particular, has been occurring with decreasing frequency. Healthcare representatives and more recently, Family Health Community Teams successfully managed to implement the Birth Plan strategy, mainly in rural communities, with the primary purpose of encouraging women and their families to take any necessary steps to give birth at the hospital. This has made it possible today for more than 90% of births to take place in hospitals, mainly belonging to the public network and the Salvadoran Institute of Social Insurance.*

In 2013, expectant mothers’ average use of MWH throughout seven MWHs was approximately 70%, which is close to the government’s goal of 80% coverage. El Salvador’s mountainous geography makes transportation difficult and medical facility accessibility challenging in many regions; transportation services are not readily supported in El Salvador, particularly in case of expectant mothers in remote areas who need to access hospitals at the impending stages before delivery. MWHs were constructed to serve the medical needs of expectant mothers from remote areas. It appears that MWHs have increased facility-based deliveries and reduced maternal and child mortality rates in remote areas.

It is clear that the UCSF and MWH systems have reduced MMR by connecting vulnerable expectant mothers to hospitals and enabling facility-based delivery [5, 6]. Since the most vulnerable group of expectant mothers are adolescents [8], the system inarguably reduced the number of maternal deaths among adolescents [4]. Similar effects were found in Nepal, where MWHs reduced maternal deaths among adolescents [23, 24]. The extremely high proportion of adolescent patients may be explained by the high rates of adolescent pregnancy in remote and rural areas [9, 25, 26], the heavy reliance of adolescents on this system, and the effective efforts of UCSF in guiding pregnant adolescents to MWHs [8-10]. However, the system was not successful in reducing pregnancy among adolescents. Adolescents were provided education about sex and maternal care once they were admitted to MWHs (therefore, not before becoming pregnant). Of course, there is some degree of sexual education in school curricula, but these efforts are evidently ineffective in reducing adolescent pregnancy [27].

One limitation of this study is that by using country-level data, we could not employ MWH as an independent variable in the regression. Cluster randomized controlled trials would be a viable solution to address this limitation. To do so, we need more granular data, such as TFR or MMR by region or village. If such data exist, then we could use the time variation as MWHs were installed across different regions and compare the MMR.

**Conclusion**

We have shown that El Salvador could reduce its MMR by improving health factors, such as total fertility rate, adolescent fertility rate, skilled birth attendance, and non-health factors, such as GDP per capita and woman empowerment. However, even considering these factors, the MMR of El Salvador decreased more than expected. We confirmed this by analyzing the residuals of a regression model. The reason for this unexplained reduction in El Salvador’s MMR appears to be attributable to health policies that not only aimed to reduce the (adolescent) fertility rate but also provided safe birthing conditions and medical services to pregnant women such as MWHs. Nevertheless, as the adolescent fertility rate is still too high and pregnancy among adolescents continues to be a significant driver of maternal deaths, more effective policies to reduce adolescent pregnancies are needed.

**List of abbreviations**

AFR: Adolescent Fertility Rate (births per 1,000 women ages 15-19); Coef: coefficient; ECOS: Equipos Comunitarios de Salud; ELECTRIC: Access to electricity, rural (% of rural population); FE: Fixed Effect; FPARLIA: Proportion of seats held by women in national parliaments (%); FPARTICI: Female Labor Force Participation %; FSECOND: School enrollment, secondary, female (% gross); GDPC: GDP per capita (dollars); HEALTH: Total health expenditure to GDP ratio (%); HEM: Hogar de Espera Materna; MDGs: Millennium Development Goals; MMR: Maternal mortality ratio (per 100,000 live births); Obs: Observation; RE: Random Effect; SKILL: Skilled Birth Attendance (% of total); TFR: Total Fertility Rate (births per woman); UCSF: Unidades Comunitarias de Salud Familiar; URBAN: Urban population (% of total); WDI: World Development Indicators;

**Declarations**

**Ethics approval and consent to participate**

This study was granted a waiver of review by the Institutional Review Board of Seoul Women’s College of Nursing (No. SWCN-201407-HR-002). The need for informed consent was waivered by the Institutional Review Board of Seoul Women’s College of Nursing.

**Consent for publication**

Not applicable.

**Availability of data and materials**

The datasets used and analyzed during the current study are available from the corresponding author.

**Competing interests**

No potential conflict of interest was reported by the authors

**Funding**

The authors report no financial or personal relationships with other people or organizations.

**Authors' contributions**

CK and HY conceived the study. CK led the analysis of all the data. HY reviewed the literature. All authors contributed to the drafting of the manuscript. All authors read and approved the final manuscript.

**Acknowledgements**

The authors would like to thank and acknowledge the work of the KOICA and Ministry of Health of El Salvador.

**References**

1. United Nations El Salvador. 3rd Report on the Progress of the Millennium Development Goals El Salvador. Government of El Salvador United Nations System in El Salvador, El Salvador; 2014. https://[file://D:/Downloads/UNDP SV Tercer-informe-ODM-ENGLISH-2014.pdf](file://D:/Downloads/UNDP%20SV%20Tercer-informe-ODM-ENGLISH-2014.pdf). Accessed 18 Oct 2021.
2. OPS. El Salvador´s Health Reform: The Right Path to Reduce Maternal Mortality. http:// 2021  [El Salvador´s Health Reform: The Right Path to Reduce Maternal Mortality (paho.org)](https://www3.paho.org/hq/index.php?option=com_content&view=article&id=10608:2015-el-salvadoras-health-reform-the-right-path-to-reduce-maternal-mortality&Itemid=39620&lang=fr). Accessed 18 Oct 2021.
3. Alkema L, Chou D, Hogan D, Zhang S, Moller A, Gemmill A, Ma Fat D, Boerma T, Temmerman M, Mathers C, Say L. Global, regional, and national levels and trends in maternal mortality between 1990 and 2015, with scenario-based projections to 2030: a systematic analysis by the UN Maternal Mortality Estimation Inter-Agency Group. Lancet 2016; 387: 462-74. http:// doi.org/10.1016/S0140-6736(15)00838-7
4. Bonawitz R, McGlasson KL, Kaiser JL, Ngoma T, Fong RM, Biemba G, Bwalya M, Hamer D, Scott NA. Quality and utilization patterns of maternity waiting homes at referral facilities in rural Zambia: A mixed-methods multiple case analysis of intervention and standard of care sites. PLoS ONE 14(11): e0225523. <https://doi.org/10.1371/journal.pone.0225523>
5. Dadi1 TL, Bekele BB, Kasaye HK, Nigussie T. Role of maternity waiting homes in the reduction of maternal death and stillbirth in developing countries and its contribution for maternal death reduction in Ethiopia: a systematic review and meta-analysis. BMC Health Services Research. 2018; 18:748-758. <https://doi.org/10.1186/s12913-018-3559-y>
6. Buser JM, Munro-Kramer M, Veliz P, Zhang X, Lockhart N, Biemba G, Ngoma T, Scott N, Lori J. How maternity waiting home use influences attendance of antenatal and postnatal care. PLoS ONE. 2021; 16(1): e0245893. <https://doi.org/10.1371/journal.pone.0245893>
7. Scott N, Kaiser JL, Vian T, Bonawitz R, Fong RM, Ngoma T, Biemba G, Boyd C, Lori JR, Hamer D, Rockers P. Impact of maternity waiting homes on facility delivery among remote households in Zambia: protocol for a quasi-experimental, mixed-methods study. BMJ Open. 2018; 8:e022224. <https://doi.org/10.1136/bmjopen-2018-022224>
8. Prado AG, Cortez R. Maternity waiting homes and institutional birth in Nicaragua: Policy options and strategic implications. International Journal of Health Planning and Management. 2012; 27: 150-166. <http://doi.org/10.1002/hpm.1107>
9. KOICA. Ex-Post Evaluation Report on Maternal and Child Health Projects in Four Latin American Countries, Seoul, 2014. <http://publication.gsis.snu.ac.kr>. Accessed 19 Oct 2021.
10. Ministerio de Salud. Redes Integrales e Integradas de los Servicios de Salud. El Salvador: Gobierno de El Salvador; 2014. <https://docplayer.es/65096340-El-modelo-de-provision-de-servicios-de-salud-redes-integrales-e-integradas-de-servicios-de-salud-el-salvador.html>. Accessed 18 Jan 2021.
11. Marroquín M. Mecanismos e incentivos para cubrir áreas desatendidas: Implementación de la estrategia de Equipos Comunitarios de Salud Familiar en El Salvador. El Salvador: Ministerio de Salud El Salvador; 2015. <https://docplayer.es/115431417-Mecanismos-e-incentivos-para-cubrir-areas-desatendidas-implementacion-de-la-estrategia-de-equipos-comunitarios-de-salud-familiar-en-el-salvador.html>. Accessed 18 Oct 2021.
12. World Health Organization. El Salvador Territorial community teams. Geneva: World Health Organization; 2018. <https://docplayer.net/148709008-El-salvador-territorial-community-teams.html>. Accessed 18 Jan 2021.
13. Portillo J. Sistema Informatico para la Dispensarizacion de los Pacientes de los Equipos Communitarios de Salud Familiar del Ministerio de Salud. San Salvador: Universidad de El Salvador; 2015. http://ri.ues.edu.sv/id/eprint/8353/.Accessed 18 Jan 2021.
14. Rodríguez M. Construyendo la esperanza Estrategias y recomendaciones en salud 2009-2014. El Salvador: Ministerio de Salud de El Salvador; 2009. <https://www.amanecersolidario.org/files/Estrategias-y-recomendaciones-en-salud-El-Salvador.pdf>. Accessed 18 Jan 2021.
15. Ministero de Salud. Lineamientos técnicos para el funcionamiento de los hogares de espera materna. El Salvador: Gobierno de El Salvador; 2017. [http://asp.salud.gob.sv/regulacion/pdf/lineamientos/lineamientos para el funcionamiento de hogares de espera materna v1.pdf](http://asp.salud.gob.sv/regulacion/pdf/lineamientos/lineamientos%20para%20el%20funcionamiento%20de%20hogares%20de%20espera%20materna%20v1.pdf). Accessed 18 Jan 2021.
16. Lori J, Wadsworth A, Munro M, Rominski S. Promoting access: The use of maternity waiting homes to achieve safe motherhood. Midwifery. 2013; 29:1095-1102. http://doi.org/10.1016/j.midw.2013.07.020
17. Chandramohan D, Cutts F, Chandra R. Effects of a maternity waiting home on adverse maternal outcomes and the validity of antenatal risk screening. International Journal of Gynaecology & Obstetrics. 1994; 46: 279-284.http:// doi.org/ 10.1016/0020-7292(94)90406-5.
18. World Development Indicators. The World Bank, Washington. 2018. http://datatopics.worldbank.org Accessed 22. Dec 2019.
19. Bishai1 DM, Cohen R, Alfonso YN, Adam T, Kuruvilla S, Schweitzer J. Factors Contributing to Maternal and Child Mortality Reductions in 146 Low- and Middle-Income Countries between 1990 and 2010. PloS ONE. 2016; 11(1):e0144908. https://doi.org/10.1371/journal.pone.0144908
20. Say L, Chou D, Gemmill A, Tunçalp Ö, Moller A, Daniels J, Gülmezoglu M, Temmerman M, Alkema L. Global causes of maternal death: a WHO systematic analysis. Lancet Glob Health. 2014; 2: e323–33. http:// doi.org/10.1016/S2214-109X(14)70227-X
21. United Nations Populations Fund. State of World Population. New York: United Nations Populations Fund; 2005. <https://www.unfpa.org/publications/state-world-population-2005>. Accessed 18 Jan 2021.
22. WHO, UNFPA. Pregnant Adolescents: Delivering on Global Promises of Hope. Geneva: WHO; 2006. <https://apps.who.int/iris/handle/10665/43368>. Accessed 18 Jan 2021.
23. Bøhler E, Rural medicine in Nepal. Tidsskr Nor Laegeforen 2010; 130(24):2506-2508. <http://doi.org/10.4045/tidsskr.10.0222>
24. Rajbhandari SP, Aryal K, Sheldon WR, Ban B, Upreti S, Regmi K, Aryal S, Winikoff B. Postpartum hemorrhage prevention in Nepal: a program assessment. BMC Pregnancy and Childbirth. 2017; 17:169. <http://doi.org/10.1186/s12884-017-1347-z>
25. Velázquez R, Pozo M. Aspectos epidemiológicos sobre adolescentes embarazadas en un área del municipio El Salvador, Guantánamo. Revista Información Cientifica. 2017; 96(3): 346-354.
26. Ruiz-Canela M, López-del Burgo C, Carlos S, Calatrava M, Osorio A, de Irala J. Familia, amigos y otras fuentes de información asociadas al inicio de las relaciones sexuales en adolescentes de El Salvador. Revista Panamerica Salud Publica. 2012; 31: 54-62.
27. Padilla de Gil M. Contribution of the Central American and Caribbean obstetrics and gynecology societies to the prevention of unsafe abortion in the region. International Journal of Gynaecology and Obstetrics. 2014; 126: S10–S12. https://doi.org /10.1016/j.ijgo.2014.03.005
