# Supplementary material for: Maternity waiting home as a potential intervention for reducing the maternal mortality ratio in El Salvador: an observational case study
Source: Arch Public Health. 2021 Dec 20;79:228. doi: 10.1186/s13690-021-00752-8 (PMC8690890; doi:10.1186/s13690-021-00752-8)
Supplement: Supplementary file 2 — Additional file 2. [file 13690_2021_752_MOESM2_ESM.docx]

**Reply to the comments of the referees**

We appreciate the useful comments given by the referees. We did our best to reflect all the comments of the referees to the revised version of the paper. Thanks to those comments, the manuscript improved a lot.

The table below is point -by-point response.

| Comments | Modifications made |
| --- | --- |
| Use of the words mixed method to avoid redundancy or repeating the same words all over. | Recognizing that the English of the manuscript was not so good, we receive professional English editing service from American Journal Experts. |
| The main concern is the study design which is not appropriate to assess the impact of maternity waiting home. | We included a new section describing the study design, as follows:  **Study design**  “Even if MWHs are important to minimizing the MMR in El Salvador, they cannot be considered in regression analysis as no data exist. In this situation, our research strategy is to run a regression using health and non-health variables that are commonly used to explain MMR and then analyze how much of the MMR reduction in El Salvador can be attributed to these variables versus how much can be attributed to other reasons that are specific to El Salvador. We can do this by analyzing the residuals, i.e., the unexplained part, in the regression.” |
| A cluster randomized controlled trial would be a better design. This should be highlighted in the discussion  and the conclusion express with more caution | One of the reviewers suggested that a cluster randomized controlled trial would be a better design and that this should be highlighted in the discussion. We accept this comment and included a paragraph pointing out the limitation of the study.  Edited as pointed out below. (p.15)  “One limitation of this study is that by using country-level data, we could not employ MWH as an independent variable in the regression. Cluster randomized controlled trials would be a viable solution to address this limitation. To do so, we need more granular data, such as TFR or MMR by region or village. If such data exist, then we could use the time variation as MWHs were installed across different regions and compare the MMR”. |
|  | In the conclusion we made more cautious interpretation of the results by saying that the reduction of El Salvador’s MMR seems to be partly attributable to the health policy including HEM as followed.  Edited as pointed out below. (p.15)  “The reason for this unexplained reduction in El Salvador’s MMR appears to be attributable to health policies that not only aimed to reduce the (adolescent) fertility rate but also provided safe birthing conditions and medical services to pregnant women such as MWHs”. |
| Suggested title “Maternity waiting home as a potential intervention for reducing maternal mortality ratio in El Salvador: an observational case study” | We changed the title as suggested:  Maternity Waiting Home as a Potential Intervention for Reducing the Maternal Mortality Ratio in El Salvador: An Observational Case Study |
| Avoid to use abbreviation in the abstract without define them before | Edited as pointed out.  We have deleted abbreviation in the abstract without defining them before. |
| What the previous existing study on the maternity waiting home? | Edited as pointed out below. (p.3)  Maternity waiting homes are residential facilities located near hospitals where pregnant women can await their delivery and be transferred to the neighboring hospital shortly before giving birth or earlier in case of complications [4]. MWH can increase the facility-based delivery rate by inducing women living in remote areas to give birth at health facilities [5, 6].  The three elements of MWHs are: i) facilities where pregnant women can comfortably reside before delivery, ii) policies and financial support, and iii) easy access to health systems with skilled personnel [7]. Despite some studies that could not find strong evidence of the effectiveness of MWHs, most studies show that MWHs have positive effects on preventing maternal death and stillbirths [5]. |
| And potentially on maternity waiting home and MMR? | Edited as pointed out below. (p.3)  In some developing countries, MWHs are an important part of national strategies to improve maternal health services [5]. In Zambia, health authorities have adopted MWHs for decades to overcome demand-side barriers and increase access to skilled birth attendants [6, 7]. |
| Describe more about the maternity waiting home. Is it a preventive strategy against MMR used in El-Salvador? | Edited as pointed out below. (p.3-4)  In El Salvador, the state of maternal health care services remains poor, particularly for the poorest populations residing in remote areas [8]. The government of El Salvador implemented the MWH program to reduce the maternal mortality ratio in rural areas [2]. |
| Is it a preventive strategy against MMR used in El-Salvador? | Edited as pointed out below. (p.2)  El Salvador is recognized as a country that has the capacity to effectively reduce its maternal mortality ratio (MMR). The Millennium Development Goals (MDG) Report states that the MMR in El Salvador reduced considerably in the 1990s and 2000s thanks to successful health reforms [1]. |
| What is the burden of MMR in El Salvador? | Edited as pointed out below. (p.2)  The next step is to provide more equitable access to health care services in remote regions and vulnerable populations [2]. |
| And tendency on the past years? in El Salvador | Edited as pointed out below. (p.2)  The MMR in El Salvador decreased from 118 to 54 per 100,000 live births from 1995 to 2015, which was the largest reduction seen among comparable Latin American countries. The average MMR of Latin American countries decreased from 118 in 1995 to 68 in 2015, a 47.2% reduction. The annual rate of MMR reduction in El Salvador was 5.2% between 1990 and 2015 [3]. |
| The study design should be clarified.  Please add more precisions on the study design, strengths and weaknesses? | As already mentioned above, we added a new section of study design.  We also included the limitations of the study: “One limitation of this study is that by using country-level data, we could not employ MWH as an independent variable in the regression. Cluster randomized controlled trials would be a viable solution to address this limitation. To do so, we need more granular data, such as TFR or MMR by region or village. If such data exist, then we could use the time variation as MWHs were installed across different regions and compare the MMR.” |
| What is of the HEM cost for one pregnant woman? And who is in charge of these costs? | We clarified this by providing more information about the cost. (p.6)  “All services provided at MWHs are free of charge. The management budget for each MWH is provided by the Department of Health even if there are cases in which a part of the budget is sponsored by regional steering committees or sponsors. During their stay at an MWH, expectant mothers are provided with meals and treatments that are particularly essential during pregnancy. After delivery, mothers are provided with clothing for their newborn babies [9, 15].” |
| What do you mean by OLS regression? | We provided explained more about ordinary least squares in p.8. |
| What about panel regressions | We explained more about panel regression in p.8.  “Panel regression with fixed effects, where time-invariant country-specific characteristics are included.  The regression model is the following:  $\log(MMR)_{it}=\alpha+\beta_{1}\log(TFR)_{it}+\beta_{2}\log(AFR)_{it}+\beta_{3}\log(SKILL)_{it}+\beta_{4}\log(HEALTH)_{it}+\beta_{5}\log(GDPC)_{it}+\beta_{6}\log(FSENCOND)_{it}+\beta_{7}\log(ELECTRIC)_{it}+\beta_{8}\log(URBAN)_{it}+\beta_{9}\log(FPARTICI)_{it}+\beta_{10}\log(FPARLIA)_{it}+(\mu_{i})+\varepsilon_{it}$  where $\mu_{i}$ is the fixed effect used only in the panel regression, which captures the effects of time-invariant country-specific characteristics.” |
| Why HEM is missing in both OLS and panel regressions? | Edited as pointed out below. (p.7)  “Even if MWHs are important to minimizing the MMR in El Salvador, they cannot be considered in regression analysis as no data exist. In this situation, our research strategy is to run a regression using health and non-health variables that are commonly used to explain MMR and then analyze how much of the MMR reduction in El Salvador can be attributed to these variables versus how much can be attributed to other reasons that are specific to El Salvador. We can do this by analyzing the residuals, i.e., the unexplained part, in the regression.” |
| What is the attributable proportion of maternity waiting home in the MMR reduction in comparison to other factors? | To explain the attributable proportion of maternity waiting home in the MMR reduction in comparison to other factors, we added Figure 2 where the contribution of other factors as well as the feasible contribution of HEM were shown.  (p.12)  “The role of the MWH program is to prevent maternal deaths as a result of pregnancy rather than to prevent the pregnancy itself. Therefore, MWH contributed to the reduction of MMR in El Salvador by providing medical service to pregnant adolescents. This effect is not reflected in the explanatory variables of the regression due to data limitations and therefore must be considered as part of the fixed effect or residuals.  In 2013, the total number of MWH patients was 2,587. Among them, 913 patients were adolescents. The share of adolescents among MWH patients was 35% (Table 3). This implies that even with preventive education, many adolescents will still become pregnant and be at risk of maternal death. Adolescents may receive MWH services and care once they are pregnant. Until now, there have been no reported maternal deaths among adolescent patients in MWHs.” |
| For proportion of reduction, please add the 95% CI | In the regression results, we substituted t-value with 95% confidence interval, as requested by the reviewer, Table 2.   |
| Result of pooled OLS regression: provide ratio (odds? Risk?) | Instead of odds ratio, we provided coefficients in pooled OLS regression. |
| Please also provide multivariate analysis relevant for confounding factors | For multivariate analysis we used OLS and panel regressions after testing whether there was multi-collinearity among the independent variables. There was no problem of multi-collinearity. |
| Panel regression: Precise the intra cluster variation? | We included the following sentence to precise the intra group variation. (p.10)  “The model explains 53.1% of the MMR variation within a country as shown by the “within” R^2^.” |
| Study of change in the MMR and users of HEM: what models did you use to assess this objective | As for the model used for the study of change in the MMR and users of HEM, we pointed out that the analysis was based on the fixed effect panel regression model. |
| Table 1. Country should be included as model's variables comparing El Salvador with other where HEM is not implemented. | Yes, country should be included as model's variables in the Table 2. Country was a unit of observation, and country fixed effects were included. |
| Table 2. Should be weighted on population size? | We did not use population size as weight, as most of the similar papers didn’t. |
| Table 3. HEM is missing in the models | As for the reason HEM was missing in both OLS and panel regressions, we explained that the regressions were run with 143 country observations, and that the data on HEM do not exist. |
| Table 4. Not clear? How can you ensure that the reduction observed is significant or not? | To make it clear, we removed Table 4, and added Figure 2, which shows the contribution of each independent variable.  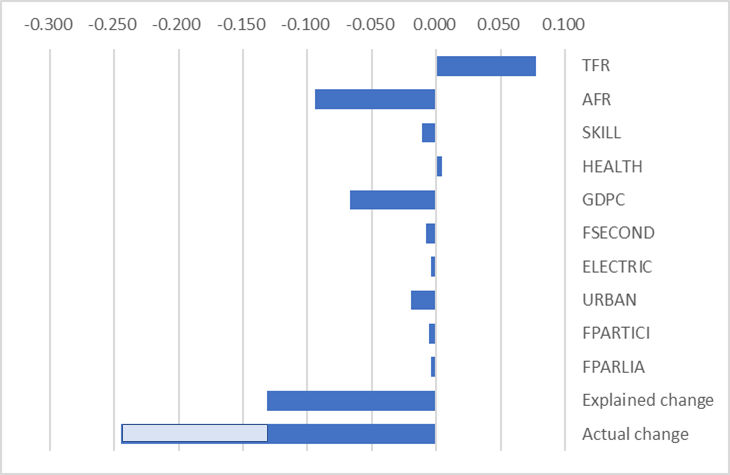 |
| Page 2 discussion line 26-27, drop how this happened? | Edited as pointed out below. (p.14)  “In 2013, expectant mothers’ average use of MWH throughout the seven MWHs was approximately 70%, which is close to the government’s goal of 80% coverage. El Salvador’s mountainous geography makes transportation difficult and medical facility accessibility challenging in many regions; transportation services are not readily supported in El Salvador, particularly in the case of expectant mothers in remote areas who need to access hospitals at the impending stages before delivery. MWHs were constructed to serve the medical needs of expectant mothers from remote areas. It appears that MWHs have increased facility-based deliveries and reduced maternal and child mortality rates in remote areas.” |
| Page 3 discussion line 18-23, provide references to support this statement. Is it only in Salvador? | Edited as pointed out below. (p.14)  “It is clear that the UCSF and MWH systems have reduced MMR by connecting vulnerable expectant mothers to hospitals and enabling facility-based delivery [5, 6]. Since the most vulnerable group of expectant mothers are adolescents [8], the system inarguably reduced the number of maternal deaths among adolescents [4]. Similar effects were found in Nepal, where MWHs reduced maternal deaths among adolescents [23, 24].” |
| References: Following references are incomplete (date of access, link etc): Ref 2, 3, 4, 5, 6, 7, 8, 13, 14, 15, 16 | We included date of access and links in the references. |
